# Oncology practitioners’ perspectives and practice patterns of post-treatment cancer survivorship care in the Asia-Pacific region: results from the STEP study

**DOI:** 10.1186/s12885-017-3733-3

**Published:** 2017-11-06

**Authors:** Raymond Javan Chan, Patsy Yates, Qiuping Li, Hiroko Komatsu, Violeta Lopez, Myat Thandar, Selva Titus Chacko, Winnie Kwok Wei So, Kanaungnit Pongthavornkamol, Myungsun Yi, Pongpak Pittayapan, Jessica Butcon, David Wyld, Alex Molassiotis, on behalf of the STEP study collaborators

**Affiliations:** 1 School of Nursing and Institute of Health and Biomedical Innovation, Queensland University of Technology, Brisbane, Australia; 2 Cancer Care Services, Royal Brisbane and Women’s Hospital, Brisbane, Australia; 3 Wuxi Medical School, Jiangnan University, Wuxi, Jiangsu China; 4 Faculty of Nursing and Medical Care, Keio University, Tokyo, Japan; 5 Alice Lee Centre for Nursing Studies, Yong Loo Lin School of Medicine, National University of Singapore, Singapore, Singapore; 6 The University of Nursing, Yangon, Myanmar; 7 College of Nursing, Christian Medical College, Vellore, India; 8 The Nethersole School of Nursing, The Chinese University of Hong Kong, Hong Kong, China; 9 Faculty of Nursing, Mahidol University, Bangkok, Thailand; 10 College of Nursing and Research Institute of Nursing Science, Seoul National University, Seoul, Republic of Korea; 11 Nursing Department of Siriraj Hospital, Mahidol University, Bangkok, Thailand; 12 College of Medicine, Bicol University, Bicol, Philippines; 13 School of Nursing, Hong Kong Polytechnic University, Hong Kong, China

**Keywords:** Cancer survivorship, Asia-Pacific region, Health professionals, Oncology practitioner, Practice patterns, Perspectives, Barriers

## Abstract

**Background:**

Most efforts to advance cancer survivorship care have occurred in Western countries. There has been limited research towards gaining a comprehensive understanding of survivorship care provision in the Asia-Pacific region. This study aimed to establish the perceptions of responsibility, confidence, and frequency of survivorship care practices of oncology practitioners and examine their perspectives on factors that impede quality survivorship care.

**Methods:**

A cross-sectional survey of hospital-based oncology practitioners in 10 Asia-Pacific countries was undertaken between May 2015–October 2016. The participating countries included Australia, Hong Kong, China, Japan, South Korea, Thailand, Singapore, India, Myanmar, and The Philippines. The survey was administered using paper-based or online questionnaires via specialist cancer care settings, educational meetings, and professional organisations.

**Results:**

In total, 1501 oncology practitioners participated in the study. When comparing the subscales of responsibility perception, frequency and confidence, Australian practitioners had significantly higher ratings than practitioners in Hong Kong, Japan, Thailand, and Singapore (all *p* < 0.05). Surprisingly, practitioners working in Low- and Mid- Income Countries (LMICs) had higher levels of responsibility perception, confidence and frequencies of delivering survivorship care than those working in High-Income Countries (HICs) (*p* < 0.001), except for the responsibility perception of care coordination where no difference in scores was observed (*p* = 0.83). Physicians were more confident in delivering most of the survivorship care interventions compared to nurses and allied-health professionals. Perceived barriers to survivorship care were similar across the HICs and LMICs, with the most highly rated items for all practitioners being lack of time, dedicated educational resources for patients and family members, and evidence-based practice guidelines informing survivorship care.

**Conclusions:**

Different survivorship practices have been observed between HICs and LMICs, Australia and other countries and between the professional disciplines. Future service planning and research efforts should take these findings into account and overcome barriers identified in this study.

**Electronic supplementary material:**

The online version of this article (10.1186/s12885-017-3733-3) contains supplementary material, which is available to authorized users.

## Background

The incidence of cancer in the Asia-Pacific region is substantial, accounting for over 30% of all cases worldwide [17]. Projected to bear the largest absolute increase in new cancer cases over the next decade, the burden of cancer in the Asia-Pacific region is expected to grow by 41% or approximately 6.5 million new cases per year [17]. The proportion of people over 65 years of age in this region is likely to double from the current 7% by 2030 [13]. This growth has significant implications for cancer services in the region across all phases of the cancer trajectory. The survivorship phase in particular has received less attention to date in health service planning. In 2005, the American Institute of Medicine (IOM) released a seminal report entitled *Lost in transition: From cancer patient to cancer survivor* [3], which recommended four essential components of survivorship care: prevention and detection of new and recurrent cancers, surveillance for cancer spread or recurrence, interventions for the physical, psychosocial and economic consequences of cancer, and treatment and coordination of care between providers. The IOM report further stipulated that quality survivorship care requires a coordinated approach by multidisciplinary practitioners including but not limited to physicians, nurses, psychologists, and social workers [3]. The STEP study (*n* = 1873) demonstrated that cancer survivors in the Asia Pacific region has significant symptom burden and unmet supportive care needs [10].

Several studies have investigated the perspectives of oncology practitioners concerning their survivorship care practice and perceived barriers that impede the implementation of quality survivorship care [1, 2, 5, 12, 14]. These studies report varying levels of standards in terms of care provision [1, 2, 5, 12, 14]. A Singaporean study of 126 multidisciplinary oncology practitioners reported there were varying levels of frequency in how often they delivered different components of survivorship care [12]. The participants in this study also reported that monitoring physical and treatment-related adverse effects is a much more prevalent practice compared monitoring psychosocial issues [12]. Such differences may be attributed to the different perceptions of the oncology practitioners regarding their responsibility, confidence levels and other barriers at the system-, practitioner- and patient- levels [12, 14]. Understanding these factors will help inform policies and targeted interventions [1, 12, 14].

Achieving high-quality cancer survivorship care requires a system level approach through implementing an effective cancer control policy as well as capacity building amongst health professionals [8]. These efforts must be evidence-based to optimise outcomes [8]. To date, most efforts to advance survivorship care have occurred in Western countries, while there has been limited research towards gaining a comprehensive understanding of survivorship care provision in the Asia-Pacific region. Therefore, the importance of cancer survivorship might not be fully appreciated in the non-Western countries in this region. With the increase in the number of cancer survivors across the Asia-Pacific region, there is a pressing need to gather evidence that can inform the design of interventions and service planning in this area.

## Methods

### Aims

This study aimed to establish the perceptions of responsibility, confidence, and frequency of survivorship care practices of oncology practitioners in relation to their provision of post-treatment survivorship care, and examine their perspectives of factors that impede quality survivorship care.

### Design

A cross-sectional survey of oncology practitioners in 10 Asia-Pacific countries was undertaken between May 2015–October 2016. The participating countries included Australia, Hong Kong, China, Japan, South Korea, Thailand, Singapore, India, Myanmar, and The Philippines. Relevant ethics and institutional research board approvals were obtained from all sites before commencement of data collection. The survey was administered via paper-based or online questionnaires. The sample size to be recruited from each participating country was based on feasibility in consultation with the respective international principal investigators. Each participating site was advised to aim for at least 100 completed questionnaires.

### Setting and sample

A principal investigator in each country was identified and asked to nominate the preferred method for recruitment. This included distribution via a national cancer care professional organisation (using email), via a specialist cancer care setting (using hard copies) that provides radiation or systemic antineoplastic therapy and/or cancer surgery, or via specialty training programs for oncology practitioners. Centres that provide cancer surgery alone were excluded. Eligible participants in this study were hospital-based oncology practitioners (i.e. physicians, nurses, allied-health professionals) who identified themselves as a care provider for patients with cancer. Inclusion criteria were: (1) aged >18 years (>21 years for Singapore); (2) spent >50% of work time on caring for patients with cancer; (3) working in a cancer care setting that provides systemic antineoplastic or radiation therapy.

### Materials

The questionnaire comprised measures of (i) demographic and work-related characteristics; (ii) three subscales that assessed oncology practitioners’ perception of responsibility (whether survivorship care is part of their role); confidence (how confident they are in delivering survivorship care), and frequency of practices (how often they provide survivorship care to patients at the completion of treatment) relevant to 29 items of survivorship care interventions. These 29 items were developed from the IOM seminal report entitled *Lost in transition: From cancer patient to cancer survivor* [3] and a review of the literature on survivorship care practices. The original version (with 17 survivorship intervention items) was piloted with oncology nurses in two Australian studies [2, 14]. For the purpose of this study, the original questionnaire used in the pilot studies was amended to include an additional 12 items to reflect a wider range of responsibilities relevant to multidisciplinary teams (Additional file 1). These items were rated as *totally disagree = 1, somewhat disagree, do not know, somewhat agree,* to *totally agree = 5* for the subscale for perception of responsibility; *cannot do at all = 0,* to *highly certain can do = 10* for the confidence subscale; and *never = 1, occasionally, often, very often,* to *all the time = 5* for the frequency subscale (Additional file 1). Additionally, (iii) individual, organisation and professional factors that impede quality survivorship care [9] were assessed using levels of agreement on a Likert scale (*not at all = 1; somewhat; quite a lot, a great deal = 4*) through a 16-item pre-determined list. Participants had an option to provide additional factors.

Principal investigators in each of the participating countries were responsible for translating the questionnaire to the language that the oncology practitioners were most proficient. A standardised translation protocol was developed as informed by the World Health Organization (WHO) [16]. For each country requiring a non-English version of the questionnaire, a forward translation was undertaken by one bilingual health professional/researcher. Secondly, a bilingual expert panel (*n* ≥ 4) was invited to confirm the content validity of the surveys. Lastly, pre-testing was undertaken by 10 oncology practitioners (wherever possible including all three disciplines: medical, nursing and allied-health) to assess face validity. At this stage, the participants were asked if anything was unclear and to provide suggestions for further amendment, with these amendments leading to the final version of the instrument used in this study.

### Data collection

The local research team or nominated clinical staff invited the potential participants via email, by post, or face-to-face in clinical areas or educational meetings. A participant information sheet was provided to the participants regardless of means of communication (email/post/face-to-face). Completion and return of the survey implied consent. For surveys distributed via professional organisations, an initial invitation was sent to the members. A reminder was sent 2 weeks after the first invitation (or at another nominated time agreed on by the professional organisation).

### Analysis

All analyses were conducted using SPSS version 22. Descriptive statistics for all outcome measures (perception of responsibility, confidence, and frequency of survivorship care provision) were calculated including means, standard deviations, and frequency distributions. We also conducted bivariate analyses (e.g. t-test, ANOVA/Kruskal-Wallis or correlation coefficients) to explore relationships between the outcome measures and a range of participant characteristics (age; gender; professional disciplines; years of experience in cancer care; practice settings; highest qualification etc.). We calculated the missing data rates for each country and tested the assumption of missing-at-random. All results were compared across all participating countries; Australia vs other countries; Low- and middle- income countries (LMICs) [Myanmar, India, Thailand, The Philippines, and China] vs High-income countries (HICs) [Japan, South Korea, Hong Kong, Singapore, Australia], defined as such according to the World Bank classifications [15]. Australian data were used as a benchmark, as existing cancer control policies in Asia-Pacific countries suggest that survivorship care in the Australian healthcare system is expected to be more developed than those of other Asia-Pacific countries.

## Results

### Participant characteristics

In total, 1501 oncology practitioners participated in the study. The majority were below 40 years of age (66.9%, *n* = 1002), female (85%, *n* = 1280), with over 6 years of experience in cancer care (63.1%, *n* = 944), oncology nurses (79.4%, *n* = 1191), had a bachelor degree or above (80.3%, *n* = 1195), working full-time (93.9%, *n* = 1410), in a main role in direct clinical care (89.5%, *n* = 1344), and working in adult care settings (81.3%, *n* = 1220). Participants were recruited from a range of settings such as tertiary referral hospitals, regional hospitals, cancer clinics and professional organisations, with the majority working in a metropolitan area (84.4%, *n* = 1263) (Table 1).

**Table 1:** Demographics and professional characteristics of the participants (*N* = 1501)

Characteristics	*N* (%)
Country	
Australia Hong Kong China Japan South Korea Thailand Singapore India Myanmar Philippines	163 (10.9) 100 (6.7) 317 (21.1) 209 (13.9) 100 (6.7) 200 (13.3) 147 (9.8) 103 (6.9) 110 (7.3) 52 (3.5)
Age	
18–29 30–39 40–49 50–59 60 and above	399 (26.6) 603 (40.3) 295 (19.7) 172 (11.5) 29 (1.9)
Years of experience in cancer care	
<1 year 1–5 years 6–10 years 11–20 years >20 years	106 (7.1) 447 (29.8) 409 (27.2) 386 (25.7) 149 (9.9)
Professional disciplines	
Physicians Nurses Allied-health	250 (16.7) 1192 (79.4) 59 (3.9)
Work status	
Full Time Part Time	1410 (93.9) 90 (6.0)
Highest qualifications	
Hospital Certificate Diploma Bachelor Degree Graduate Diploma/Certificate Master Doctorate/Doctor of Medicine	37 (2.5) 256 (17.2) 666 (44.8) 194 (13.0) 230 (15.5) 105 (7.1)
Work settings	
Public Private Mixed	958 (63.9) 446 (29.8) 95 (6.3)
Patient setting	
Adults Paediatrics Mixed	1220 (81.4) 22 (1.5) 256 (17.1)
Geographical location of workplace	
Metropolitan Regional Rural Mixed	1263 (84.4) 76 (5.1) 23 (2.3) 124 (8.3)

### Scale reliability and missing data

Cronbach’s alpha for all subscales (perception of responsibility, frequency, confidence and impeding factors) ranged between 0.92 to 0.97. Using the spearman *r* correlation coefficients, there were statistically significant correlations between all subscales of perception of responsibility, confidence and frequency, further supporting the internal consistency of the scale. With regards to missing data, less than 2% of samples had missing data for all countries except for Australia (14.7%, *n* = 24). The Australian participants who had a managerial or administrative role were more likely to have missing data (*p* < 0.05).

### Outcome measures and demographic and work-related characteristics

There was a weak positive correlation between age and all four confidence subscales (all *p < 0.001*). No statistically significant correlation was found between age and the responsibility perception and frequency subscales. Participants with a bachelor degree or above had higher confidence scores for all four subscales (all *p < 0.001*), and higher frequency scores for the surveillance and coordination subscales (all *p < 0.001*) than those with lower levels of education. Those working in the public settings had higher confidence scores for all subscales (all, *p < 0.001*), higher responsibility perception scores for the prevention subscale (*p* < 0.05) and higher frequency scores for the prevention (*p* < 0.05) and coordination (*p* < 0.005) subscales compared to those working in the private or mixed settings. Those working in non-metropolitan areas had significantly higher scores for *all* subscales (i.e. responsibility perception, confidence and frequency, all *p < 0.001*), except for responsibility perception -surveillance subscale.

### Comparisons between Australia and other countries

When comparing all subscales including responsibility perception, frequency and confidence between Australia and other countries, Hong Kong, Japan, Thailand, and Singapore had significantly lower scores across majority of the subscales (all *p* < 0.05) than Australia (Table 2). China, India, Myanmar and The Philippines achieved either significantly higher scores (all p < 0.05) or similar scores (i.e. not significant differences) compared to Australia for the majority of the subscales. South Korea had significantly lower ratings for six subscales (*p < 0.05*) and had either significantly higher (*p < 0.005*) or similar ratings for six subscales.

**Table 2:** Comparisons between Australia and all countries using independent-samples *t*-tests (*N* = 1501)

	Possible range	Australia	Hong Kong	China	Japan	South Korea	Thailand	Singapore	India	Myanmar	Philippines
		*N* = 163 (Ref)	*N* = 100	*N* = 317	*N* = 209	*N* = 100	*N* = 200	*N* = 147	*N* = 103	*N* = 110	*n* = 52
Perception of responsibility											
Prevention	2–10	7.58 (2.14)	6.79 (1.95)**	8.88 (1.60) **	6.55 (1.84)**	NS	6.40 (1.9)**	6.64 (2.4)**	8.33 (2.05)**	NS	6.67 (2.87)*
Intervention	14–70	62.08 (7.19)	54.38 (10.35)**	NS	58.74 (6.88)**	57.65 (9.95)**	49.59 (13.39)**	55.82 (10.67)**	NS	51.37 (9.70)**	53.94 (11.73)**
Surveillance	4–20	14.83 (4.00)	NS	18.61 (2.19)**	16.16 (2.89)**	17.07 (3.30)**	NS	15.79 (3.76)*	17.78 (3.08)**	NS	NS
Coordination	9–45	39.14 (5.81)	36.07 (6.95)**	40.49 (5.62)*	35.39 (6.10)**	37.57 (6.57)*	31.45 (9.96)**	NS	NS	33.65 (6.30)**	NS
Confidence											
Prevention	0–20	10.21 (4.97)	7.86 (4.40)**	12.47 (5.03)**	3.88 (3.93)**	NS	12.89 (4.41)**	6.84 (5.02)**	12.47 (4.06)**	12.08 (5.22)**	NS
Intervention	0–140	85.28 (17.93)	64.64 (15.20)**	77.31 (19.82)**	51.22 (18.46)**	67.83 (16.39)**	67.59 (17.86)**	54.45 (23.73)**	77.46 (14.52)**	72.11 (24.56)**	70.80 (25.63)**
Surveillance	0–40	25.12 (8.98)	22.48 (7.67)*	29.25 (8.28)**	16.99 (9.13)**	NS	27.46 (7.62)*	18.68 (10.16)**	30.31 (5.88)**	28.82 (9.83)**	NS
Coordination	0–90	70.26 (16.29)	57.33 (13.72)**	63.69 (18.47)**	34.65 (20.50)**	58.08 (13.71)**	49.50 (17.85)**	50.81 (21.06)**	NS	63.61 (21.12)*	NS
Frequency											
Prevention	2–10	4.84 (2.21)	4.19 (1.61)*	6.08 (2.15)**	3.01 (1.21)**	NS	3.51 (1.81)**	3.52 (1.62)**	6.07 (1.92)**	5.94 (1.86)**	NS
Intervention	14–70	46.87 (11.61)	38.71 (8.77)**	NS	32.62 (9.81)**	37.78 (9.35)**	30.62 (9.82)**	34.21 (10.46)**	NS	41.31 (10.92)**	NS
Surveillance	4–20	10.80 (4.42)	10.18 (3.5)**	14.73 (3.96)**	8.78 (3.51)**	NS	10.75 (4.38)**	8.73 (3.70)**	15.02 (3.18)**	14.14 (3.53)**	13.9 (5.73)**
Coordination	9–45	29.55 (8.59)	26.38 (6.99)**	NS	18.22 (7.01)**	25.42 (8.29)**	NS	24.28 (8.19)**	33.15 (6.62)**	NS	36.73 (7.54)**

*Note*. All subscales: higher scores represent higher levels of responsibility perception, higher levels of confidence and higher frequency of care delivery; *p < 0.05; **p < 0.005

### Comparisons between professional disciplines

For most of the responsibility perception, confidence and frequency subscales, there were significant differences amongst physicians, nurses and allied-health professionals (all *p < 0.001*) (Table 3). In terms of responsibility perception, the *post-hoc* tests indicate physicians and nurses had significantly higher ratings for the prevention and surveillance subscales than allied-health professionals (both *p < 0.001*). Nurses had significantly higher ratings for the intervention and coordination subscales than physicians (*p < 0.001*) and allied-health professionals (*p < 0.05*). Concerning confidence and frequency, compared to nurses and allied-health professionals, physicians also had significantly higher levels of confidence in delivering all survivorship care including prevention (*p < 0.001*), intervention (*p < 0.001*), surveillance (*p < 0.001*) and coordination (*p < 0.05*) and had significantly higher frequency scores for prevention, surveillance and coordination (*p < 0.001*), but not statistically significant for intervention.

**Table 3:** Relationships between professional disciplines and perception of responsibility, levels of confidence, frequency of survivorship care practice using analysis of variance

		Number	M (SD)	Possible range	F(df)	*P*
Perception of responsibility						
Prevention	Physicians Nurses Allied-health	245 1169 59	7.48 (2.27) 7.53 (2.08) 5.76 (2.78)	2–10	19.09 (1497)	<0.001
Intervention	Physicians Nurses Allied-health	245 1169 59	52.21 (14.07) 58.83 (8.83) 48.90 (12.37)	14–70	65.48 (1470)	<0.001
Surveillance	Physicians Nurses Allied-health	258 1180 59	17.50 (3.10) 16.47 (3.42) 11.85 (5.50)	4–20	63.24 (1486)	<0.001
Coordination	Physicians Nurses Allied-health	247 1171 59	33.99 (10.24) 38.09 (6.11) 31.42 (10.75)	9–45	54.72 (1474)	<0.001
Confidence						
Prevention	Physicians Nurses Allied-health	247 1173 59	13.72 (4.70) 9.31 (5.50) 6.98 (5.67)	0–20	77.50 (1476)	<0.001
Intervention	Physicians Nurses Allied-health	245 1162 58	75.82 (17.59) 67.97 (22.32) 54.50 (26.35)	0–140	22.61 (1462)	<0.001
Surveillance	Physicians Nurses Allied-health	248 1165 58	31.72 (5.91) 24.08 (9.74) 15.72 (10.73)	0–40	100.31 (1468)	<0.001
Coordination	Physicians Nurses Allied-health	243 1163 58	60.40 (20.47) 26.43 (20.94) 45.50 (24.22)	0–90	12.12 (1461)	<0.001
Frequency						
Prevention	Physicians Nurses Allied-health	244 1169 58	5.44 (2.44) 4.54 (2.14 3.60 (2.01)	2–10	23.95 (1468)	<0.001
Intervention	Physicians Nurses Allied-health	243 1154 58	41.10 (13.01) 40.17 (12.05) 33.53 (12.30)	14–70	9.18 (1452)	<0.001
Surveillance	Physicians Nurses Allied-health	246 1166 57	15.1 (2.93) 11.19 (4.50) 8.70 (4.78)	4–20	113.4 (1466)	<0.001
Coordination	Physicians Nurses Allied-health	220 1161 56	30.29 (8.26) 26.48 (8.90) 22.29 (10.72)	9–45	24.46 (1434)	<0.001

*Note*. All subscales: higher scores represent higher levels of responsibility perception, higher levels of confidence and higher frequency of care delivery

### Comparisons between LMICs and HICs

There were significant differences in the ratings between those who worked in LMICs and HICs. Practitioners working in LMICs had higher levels of responsibility perception, confidence and frequencies of delivering survivorship care than those working in HICs (*p < 0.001*) (Table 4). These differences were consistently observed except for the responsibility perception of care coordination (*p = 0.83*).

**Table 4:** Low- and middle- income countries vs. high income countries perceived responsibilities, levels of confidence, frequency of survivorship care practice

				Number	Total score M (SD)	Possible range	t (df)	*P* value
Perception of responsibility								
	Prevention		HICs LMICs	719 782	7.85 (2.11) 7.02 (2.14)	2–10	−7.58 (1499)	<0.001
	Intervention		HICs LMICs	700 774	58.17 (9.14) 56.57 (11.49)	14–70	2.97 (1449)	.003
	Surveillance		HICs LMICs	707 781	15.74 (3.60) 17.11 (3.50)	4–20	−7.42 (1486)	<0.001
	Coordination		HICs LMICs	701 777	37.18 (6.49) 37.09 (8.20)	9–45	.21 (1452)	.830
Confidence								
	Prevention		HICs LMICs	701 779	7.27 (5.08) 12.36 (5.05)	0–20	−19.3 (1478)	<0.001
	Intervention		HICs LMICs	698 768	63.26 (22.81) 73.72 (20.30)	0–140	−9.24 (1401)	<0.001
	Surveillance		HICs LMICs	695 777	20.95 (9.51)) 28.69 (8.66)	0–40	−16.25 (1411)	<0.001
	Coordination		HICs LMICs	695 770	51.89 (22.21) 60.93 (19.19)	0–90	−8.29 (1379)	<0.001
Frequency								
	Prevention		HICs LMICs	695 777	3.87 (1.82) 5.34 (2.33)	2–10	−13.61 (1444)	<0.001
	Intervention		HICs LMICs	684 772	37.48 (11.40) 42.32 (12.62)	14–70	−7.70 (1453)	<0.001
	Surveillance		HICs LMICs	692 778	9.76 (3.93) 13.61 (4.40)	4–20	−17.74 (1470)	<0.001
	Coordination		HICs LMICs	690 748	24.01 (8.81) 29.55 (8.55)	9–45	−12.11 (1436)	<0.001

*Note*. All subscales: higher scores represent higher levels of responsibility perception, higher levels of confidence and higher frequency of care delivery; *HICs* high income countries; *LMICs* low- and middle-income countries

### Barriers that impede quality survivorship care

Participants identified a number of barriers that impede quality survivorship care (Table 5). These barriers were similar across the HICs and LMICs, with the most highly rated items for the total sample being lack of time, dedicated educational resources for patients and family members, and evidence-based practice guidelines informing survivorship care. Overall, lack of time and communication barriers between the practitioners and family members were rated to be the top barrier by the participating countries except for Australia and India which rated ‘no end of treatment consultation’ and ‘don’t know what survivorship care is’ to be the top barrier, respectively.

**Table 5:** Top five perceived factors that impedes quality survivorship care

	Australia	Hong Kong	China	Japan	South Korea	Thailand	Singapore	India	Myanmar	Philippines	HICs	LMICs
*N*=	138	100	317	208	100	200	147	103	110	52	693	782
Individual/Professional Level												
Don’t know what survivorship care is				✓				✓				
Lack time	✓	✓	✓	✓	✓		✓	✓			1	1
Lack knowledge/ skills		✓		✓			✓				2	
Don’t know where the patient is at in their disease trajectory				✓								
Communication barriers between you and the patient				✓		✓			✓	✓		
Communication barriers between you and the family members						✓			✓	✓		
Family members lack of interest	–	–	–	–	–	–	–	–	–	✓		–
Organisational Level												
Survivorship care is not a priority for my organisation			✓									
Lack an appropriate physical location (e.g. a quiet room)			✓		✓	✓		✓	✓			4
No end of treatment consultation dedicated to survivorship care in my organisation	✓		✓					✓	✓		5	2
Lack of evidence-based practice guidelines informing survivorship care	✓	✓	✓		✓		✓					3
Lack of dedicated educational resources for patients	✓	✓			✓	✓	✓	✓	✓	✓	4	5
Lack of dedicated educational resources for family members	✓	✓			✓	✓					3	

*Note*. ✓- Top five factors that impedes quality survivorship care (only the items that received at least one top five rating were included); *HICs* high income countries; *LMICs* low– and middle- income countries

## Discussion

To the best of our knowledge, this is the largest study that examined oncology practitioners’ perspectives on survivorship practices in the Asia-Pacific region and is the first international study that compared survivorship practices between LMICs and HICs. Although the intervention items examined in the questionnaire comprise the essential components of quality survivorship care, there were varying levels of agreement among oncology practitioners in terms of their responsibility for covering these components of care.

This study identified significant associations between perception of responsibility, confidence and frequency of care. The more the participants agreed the care should be part of their role, the more confident they were and the more frequently they delivered the care. These relationships were not confirmed by previous work with a smaller sample size [14]. The findings of the present study highlight the importance of having clearly defined roles and responsibilities within the multidisciplinary team in relation to survivorship care. Effective communication within the multidisciplinary team to ensure everyone is well aware of their roles is critical for ensuring patients’ needs do not go undetected or unmanaged. These recommendations are consistent with previous qualitative findings with oncology nurses [7].

Using Australia as a benchmark, participants in Australia had higher ratings compared to Hong Kong, Japan, Thailand and Singapore; but not China, South Korea, Myanmar, India and The Philippines, with the latter countries demonstrating similar or higher ratings than Australia. Further qualitative research will be required to investigate the differences between these countries. Reasons for these discrepancies among the Asia-Pacific countries are likely multifactorial. The countries have different cancer control policies that may or may not have an emphasis on the post-treatment phase of care, and are at different stages of development in terms of delivery of comprehensive cancer services. The differences are also likely to be due to differences in cancer workforce profiles. For example, although the Singaporean National Cancer Centre documented the importance of survivorship care as early as 2005 [11], the uptake of survivorship care provision among nurses has been slow due to workforce shortages. Such shortages lead to intense clinical workload and nurses’ perception of not having sufficient time to deliver survivorship care even though they reported that they have the responsibility to do so.

Physicians, nurses and allied-health professionals differed in their perspectives of their role, confidence levels and frequency of survivorship care. This finding is consistent with other research reporting the differences in the patterns of follow-up practices between physicians and other practitioners such as nurses and allied-health professionals [12]. The physicians in our study consistently reported higher levels of confidence in the delivery of survivorship care than nurses and allied-health professionals. Professional development programs can be developed to enhance the knowledge and confidence of all oncology practitioners in their provision of survivorship care.

The differences between the ratings of those from LMICs and HICs, with the LMICs having higher ratings in perception of responsibility, confidence and frequencies of care were unexpected. No previous study has examined these outcomes. This finding may be explained by the the specialization of oncology practitioners in HICs. That is, in many HICs, routine referral of patients to various specialists to care for specific issues faced by patients is common. For example, patients in HICs might be referred to a sexual health concern to a sex counsellor, whereas practitioners in the LMICs would normally assume the care responsibility without such resources available. A second possibility is that practitioners in the HICs could have higher care expectations for their healthcare systems and patients’ standards of care, which could have contributed to a sense of diminished confidence. Further research will be required to gain an in-depth understanding of these differences.

In this study, we highlighted a number of barriers that prevent oncology practitioners from delivering quality survivorship care. It is important that future service planning addresses these barriers. Lack of time has been repeatedly reported in the literature as a barrier to implementation of quality survivorship care [4, 14]. Much advocacy work will be required to ensure health policies will be developed to not only raise the importance of cancer survivorship care, but also to ensure the cancer care workforce will be given adequate resources for providing quality survivorship care, especially in the ambulatory care setting. Given that the lack of educational patient resources available is a notable barrier, HICs have the responsibility to share resources with LMICs. Evidence-based, consumer informed resources for survivors who speak languages other than English, such as Mandarin, Cantonese, Vietnamese are already available in Australia [6]. These resources may be adapted to suit the cultural contexts for various countries in this region. A coordinated approach for building cancer survivorship capacity in this region should minimize duplicate efforts by involving oncology survivorship experts in the HICs. It would be opportunistic for organisations such as the WHO to develop policies and strategic plans in collaboration with countries in this region.

### Limitations

This study has several limitations. First, this study used a convenience sampling method, which does not allow us to calculate response rates to support the representability of our findings. Therefore, we were unable to establish the characteristics of the oncology workforce in all participating sites and countries. Second, the majority of participants were nurses. Physicians and allied-health were under-represented in this sample. However, such proportions are commonly seen in other multidisciplinary surveys of the same nature [12]. Third, we were unable to compare data across care settings (i.e. tertiary referral hospitals, regional centres, university hospitals or professional organisations) where participants were recruited. The standards and criteria for each of these settings vary significantly across the participating countries. Despite these limitations, this study is the first to gain a comprehensive understanding of practice patterns and perspectives of oncology practitioners in this region. The findings from this study are critical for informing service planning and capacity building activities in the Asia-Pacific region.

## Conclusions

Different survivorship practices have been observed between HICs and LMICs, Australia and other countries and between the professional disciplines. Future service planning and research efforts should take these findings into account and overcome barriers identified in this study.

## Additional file

Additional file 1: Survivorship care items included in the questionnaire. (DOC 44 kb)

## Abbreviations

HICs: High-Income Countries
IOM: Institute of Medicine
LMIC: Low- and Mid- Income Countries
WHO: World Health Organization
