# Correction to: Oncology practitioners’ perspectives and practice patterns of post-treatment cancer survivorship care in the Asia-Pacific region: results from the STEP study

**DOI:** 10.1186/s12885-018-4105-3

**Published:** 2018-03-01

**Authors:** Raymond Javan Chan, Patsy Yates, Qiuping Li, Hiroko Komatsu, Violeta Lopez, Myat Thandar, Selva Titus Chacko, Winnie Kwok Wei So, Kanaungnit Pongthavornkamol, Myungsun Yi, Pongpak Pittayapan, Jesson Butcon, David Wyld, Alex Molassiotis, on behalf of the STEP study collaborators

**Affiliations:** 1 School of Nursing and Institute of Health and Biomedical Innovation, Queensland University of Technology, Brisbane, Australia; 2 Cancer Care Services, Royal Brisbane and Women’s Hospital, Brisbane, Australia; 3 Wuxi Medical School, Jiangnan University, Wuxi, Jiangsu China; 4 Faculty of Nursing and Medical Care, Keio University, Tokyo, Japan; 5 Alice Lee Centre for Nursing Studies, Yong Loo Lin School of Medicine, National University of Singapore, Singapore, Singapore; 6 The University of Nursing, Yangon, Myanmar; 7 College of Nursing, Christian Medical College, Vellore, India; 8 The Nethersole School of Nursing, The Chinese University of Hong Kong, Hong Kong, China; 9 Faculty of Nursing, Mahidol University, Bangkok, Thailand; 10 College of Nursing and Research Institute of Nursing Science, Seoul National University, Seoul, Republic of Korea; 11 Nursing Department of Siriraj Hospital, Mahidol University, Bangkok, Thailand; 12 College of Medicine, Bicol University, Bicol, Philippines; 13 School of Nursing, Hong Kong Polytechnic University, Hong Kong, China

## Correction

It has been highlighted that the original manuscript [1] contains a typesetting error in the name of Jesson Butcon. This was incorrectly captured as Jessica Butcon in the original manuscript which has since been updated.
