# Supplementary material for: Oncology practitioners’ perspectives and practice patterns of post-treatment cancer survivorship care in the Asia-Pacific region: results from the STEP study
Source: BMC Cancer. 2017 Nov 6;17:715. doi: 10.1186/s12885-017-3733-3 (PMC5674781; doi:10.1186/s12885-017-3733-3)
Supplement: Additional file 1: — Survivorship care items included in the questionnaire. (DOC 44 kb) [file 12885_2017_3733_MOESM1_ESM.doc]

**Additional file 1** Survivorship care items included in the questionnaire

| Prevention of recurrent and new cancers, and other late effects | For each survivorship care items, three questions below were asked:  1. The levels of agreement amongst health professionals on whether the care should be their responsibility (*likert*)   - 1- Totally disagree - 2- Somewhat disagree - 3- Don't know - 4- Somewhat agree - 5- Totally agree   2. The levels of confidence of health professionals in delivering the care (Numeric analogue scale)   - 0- Cannot do at all - 5- Moderately can do - 10- Highly certain can do   3. The levels of frequency of providing survivorship  care by health professionals (*likert*)   - 0- Never 1- Occasionally - 2- Often - 3- Very often - 4- All of the time |
| --- | --- |
| 1. Discuss information on genetic counselling and testing to identify high risk individuals who could benefit from more comprehensive cancer surveillance, chemoprevention, or other risk reducing treatment |
| 1. As appropriate, discussing information on known effective chemoprevention strategies for secondary prevention |
| Intervention for physical and psychosocial consequences of cancer and its treatment |
| 1. Discussing patient/family peer support groups |
| 1. Conducting distress screening for psychological risks |
| 1. Discussing psychological side effects (ie. Emotional problems, adjustment issues, anxiety, depression etc) |
| 1. Discussing and managing intimacy and sexuality issues |
| 1. Discussing and managing pain |
| 1. Discussing and managing fertility issues |
| 1. Discussing and managing long-term physical side effects/late effects (ie. Memory problems, trouble sleeping, fatigue, difficulty concentrating, peripheral neuropathies, hot flushes, etc) |
| 1. Providing health education to survivors regarding their diagnoses, treatment exposures, and potential late and long-term effects |
| 1. Discussing and encouraging appropriate exercise and physical activity |
| 1. Providing healthy diet recommendations including alcohol consumption |
| 1. Encouraging health behaviours including sunscreen use, smoking and alcohol consumption |
| 1. Discussing and managing parenting and other help at home |
| 1. Discussing and managing employment and financial consequences of cancer |
| 1. Providing resources to assist with financial and insurance issues |
| Surveillance for cancer recurrence |
| 1. Providing information about how to identify signs of cancer spreading or recurrence |
| 1. Carrying out medical check-ups at follow-up, including taking history |
| 1. Providing screening recommendations for second cancers; periodic testing and examination, and the schedule on which they should be performed |
| 1. Addressing psychological impacts from their fear of cancer recurrence/relapse |
| Coordination of care to ensure that all the survivor’s health needs are met |
| 1. Ensuring linkage with appropriate external supportive services |
| 1. Providing information on who to contact with questions and problems |
| 1. Communicating the survivorship care provided with the rest of the healthcare team |
| 1. Communicating the survivorship care provided with the patient’s primary healthcare providers |
| 1. Organising/Ensuring the patient has a schedule of follow-up appointments with the cancer care doctors |
| 1. Providing referrals to specialists and resources as indicated |
| 1. Empowering survivors to advocate for their own healthcare needs |
| 1. Using treatment summaries and/or care plans |
| 1. Ensuring the patient has a schedule of follow-up appointments with primary healthcare providers |
